# Wildlife conservation and management in China: achievements, challenges and perspectives

**DOI:** 10.1093/nsr/nwab042

**Published:** 2021-03-13

**Authors:** Guangping Huang, Xiaoge Ping, Weihua Xu, Yibo Hu, Jiang Chang, Ronald R Swaisgood, Jiang Zhou, Xiangjiang Zhan, Zejun Zhang, Yonggang Nie, Jie Cui, Michael Bruford, Zhihe Zhang, Baoguo Li, Li Zhang, Zhi Lv, Fuwen Wei

**Affiliations:** CAS Key Laboratory of Animal Ecology and Conservation Biology, Institute of Zoology, Chinese Academy of Sciences, China; Endangered Species Scientific Commission of the People's Republic of China, China; State Key Laboratory of Urban and Regional Ecology, Research Center for Eco-Environmental Sciences, Chinese Academy of Sciences, China; CAS Key Laboratory of Animal Ecology and Conservation Biology, Institute of Zoology, Chinese Academy of Sciences, China; Center for Excellence in Animal Evolution and Genetics, Chinese Academy of Sciences, China; State Key Laboratory of Environmental Criteria and Risk Assessment, Chinese Research Academy of Environmental Sciences, China; Institute for Conservation Research, San Diego Zoo Global, USA; School of Karst Sciences, Guizhou Normal University, China; CAS Key Laboratory of Animal Ecology and Conservation Biology, Institute of Zoology, Chinese Academy of Sciences, China; Center for Excellence in Animal Evolution and Genetics, Chinese Academy of Sciences, China; Key Laboratory of Southwest China Wildlife Resources Conservation (Ministry of Education), China West Normal University, China; CAS Key Laboratory of Animal Ecology and Conservation Biology, Institute of Zoology, Chinese Academy of Sciences, China; Center for Excellence in Animal Evolution and Genetics, Chinese Academy of Sciences, China; CAS Key Laboratory of Molecular Virology & Immunology, Institute Pasteur of Shanghai, Chinese Academy of Sciences, China; Sustainable Places Research Institute, Cardiff University, UK; Sichuan Academy of Giant Panda, China; Shaanxi Key Laboratory for Animal Conservation, Northwest University, China; Key Laboratory for Biodiversity and Ecological Engineering of Ministry of Education, College of Life Sciences, Beijing Normal University, China; Center for Nature and Society, College of Life Sciences, Peking University, China; CAS Key Laboratory of Animal Ecology and Conservation Biology, Institute of Zoology, Chinese Academy of Sciences, China; Center for Evolution and Conservation Biology, Southern Marine Science and Engineering Guangdong Laboratory (Guangzhou), China; Endangered Species Scientific Commission of the People's Republic of China, China; Center for Excellence in Animal Evolution and Genetics, Chinese Academy of Sciences, China

Dramatic global changes to the environment have wrought unprecedented reductions in biodiversity, with >28% species assessed by the International Union for Conservation of Nature (IUCN) under the threat of extinction [1]. China, as one of the world's megadiverse countries, plays a critical role in global biodiversity conservation. The upcoming 15th Conference of Parties (COP15) for Convention on Biological Diversity (CBD) serves as an opportunity for China to make ambitious but pragmatic commitments to elevate its wildlife conservation and management activities. The development of an ‘Ecological Civilization’, which endorses the ancient Taoist idea of Unity of Nature and Man (UNM) to achieve harmony between human beings and nature, has greatly facilitated the mainstreaming of biodiversity conservation in China [2]. Experiences from China will shed light on wildlife conservation worldwide.

## STATUS AND ACHIEVEMENTS

### Species recovery and habitat protection

**Figure 1.**
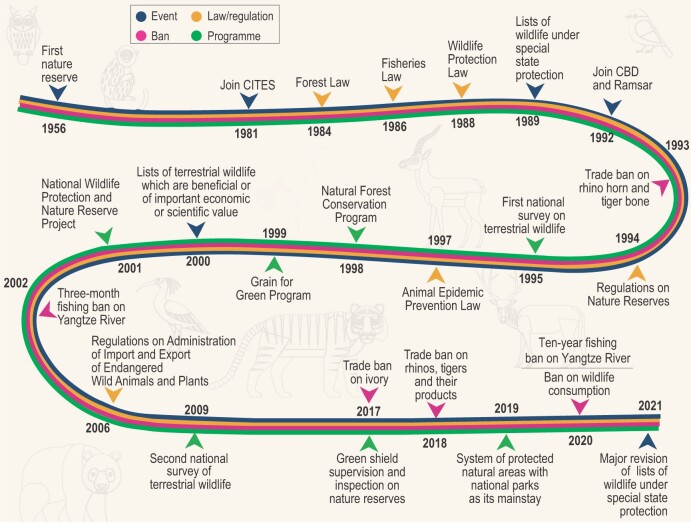
Timeline of laws/regulations (orange), national programs (green), bans (purple) and big events (blue) related to wildlife conservation and management in China.

Wildlife in China are protected and managed by different levels of administrations according to their classification and grading. The departments of forestry and grassland, and fisheries administration under the State Council shall be responsible for the nationwide administration of terrestrial and aquatic wildlife, respectively (Table 1). Beginning with the establishment of the first nature reserve in 1956, China took significant measures to protect its wildlife, including the promulgation and revision of laws and regulations, establishment of a legal framework with specific species listed according to endangerment or ecological, scientific or social value, signing international conventions and multilateral agreements, and implementing national bans, projects and surveys (Fig. 1). Flagship species, such as the giant panda, big cats, snub-nosed monkeys, gibbons, dolphins and ungulates, are afforded additional protection. After the outbreak of COVID-19, China's top legislature immediately made the decision to ban wildlife consumption throughout the country, crack down on illegal wildlife trade and promote environmental protection. Subsequently, the government updated the National Catalogue of Livestock and Poultry Genetic Resources in 2020, and Lists of Wildlife Under Special State Protection in 2021, with the number of protected species increasing from ∼500 to ∼1500, covering over 20% more threatened species (Table 1).

**Figure 2.**
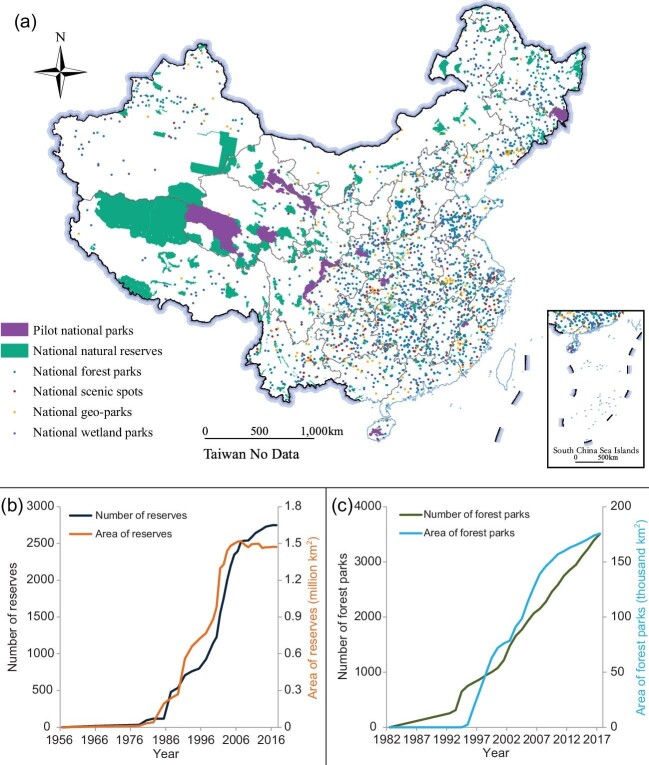
Distribution, area and number growth of major types of protected areas in China. Distribution of major types of protected areas (a); area and number growth of nature reserves (b) and forest parks (c) with data from reference [4].

This direct regulatory protection of wildlife is supplemented by a large portfolio of measures designed to protect habitats in support of wildlife. Although China had a relatively late start in developing its Protected Area (PA) system, it has already surpassed the Aichi Target 11 of 17% of terrestrial and inland water. By the end of 2018, China had established 11 800 PAs, covering ∼18% of its entire land surface (Fig. 2a) and 89% of species under special state protection [3].

Nature reserves are afforded the highest level of protection and have increased rapidly from 1990 to 2007, currently covering ∼15% of the land surface (Fig. 2b). Other types of PAs—including scenic spots, forest parks, geological parks, wetland parks and desert parks—have also been established to meet multiple conservation goals (Fig. 2c) and are integrated with the PA system to protect China's natural heritage in a wider sense [4].

China supplements habitat protection with other measures such as eco-restoration and eco-compensation. Two of the world's largest initiatives—the Natural Forest Conservation Program and the Grain for Green Program, provide economic incentives for human communities to protect and restore habitats in support of wildlife conservation. Moreover, these *in situ* efforts have been supported by a growing *ex situ* conservation portfolio advanced by the establishment of >240 zoos and ∼250 breeding centers [3]. Several reintroduction projects have been successfully established for the giant panda, Pere David's deer, crested ibis and Chinese alligator. National wildlife monitoring networks have been established, and national evaluation of threatened species has been implemented, which greatly improve the scientific basis for decision-making. Considering the human impact on freshwater and oceanic biodiversity, China has implemented a 10-year fishing ban in pivotal waters of the Yangtze River and summer fishing moratorium in oceans.

**Table 1.** Number of animal species included in old (1989) and updated (2021) Lists of Wildlife Under Special State Protection (WSSP) and threatened species in China Biodiversity Red List (CBRL) included in WSSP^a^.

							CBRL threatened species included	
	WSSP class I		WSSP class II		Total		in WSSP [number (ratio)]^b^	
Taxa	Old	Updated	Old	Updated	Old	Updated	Old	Updated
Mammal	68	99	90	86	158	185	109 (61.24%)	113 (63.48%)
Bird	43	92	204	302	247	394	79 (54.11%)	137 (93.84%)
Reptile	6	19	11	83	17	102	18 (13.14%)	59 (43.07%)
Amphibian	0	7	17	86	17	93	9 (5.11%)	66 (37.5%)
Fish	4	10	11	149	15	159	13 (4.41%)^c^	61 (20.68%)^c^
Invertebrate	13	14	25	480	38	494		
**Total**	**134**	**241**	**358**	**1186**	**492**	**1427**	**228 (24.46%)**	**436 (46.78%)**

^a^Data source: CBRL; CITES Appendices in China (Species + and Chinese Version of CITES Appendices (enter into force on 26 November 2019)); Ping & Zeng (2020) Changes in nomenclature of animals included in Lists of Wild Animals under Special State Protection in China and impacts on wildlife conservation. *Scientia Sinica Vitae***50**: 33–43 [16]. ^b^Threatened species in CBRL included those species assessed as Critically Endangered, Endangered and Vulnerable. ^c^Only freshwater fishes are included in CBRL.

With the implementation of these national laws, PAs and conservation projects (Fig. 1), a number of species have experienced population increases and habitat expansion, and the national threatened categories of >100 mammalian species have been downgraded [5]. Some flagship species such as the giant panda, snow leopard, Tibetan antelope and crested ibis, have recovered from the brink of extinction, and been downgraded from ‘Endangered’ to ‘Vulnerable’ or from ‘Critically Endangered’ to ‘Endangered’ by the IUCN.

### Combating illegal wildlife trade

Significant headway has been made recently in combating illegal wildlife trade in China. To further mitigate impacts on Chinese wildlife from international trade, China has proposed adding endemic species for protection under the Convention on International Trade in Endangered Species of Wild Fauna and Flora (CITES), providing additional protections for ∼830 animal species. A trade ban on tiger bones and rhino horns has been implemented since 1993 (Fig. 1). The last few years have witnessed a large shift in regulatory activity, with several trade-related regulations and enforcements established (Fig. 1): wholescale bans on elephant ivory and all rhino and tiger products have recently been supplemented with a suspended trading on live wildlife during the outbreak of COVID-19. The Inter-Ministerial Joint Conference for Combating Illegal Wildlife Trade, comprising 27 ministries in 2020, was established to combat illegal wildlife trade, indicating China's intolerance of wildlife trafficking and determination to crack down on the trade. Moreover, China has increased collaboration with other nations to counter wildlife trafficking, and to form multilateral wildlife law enforcement operations. In 2014, a law enforcement activity dubbed ‘Cobra II’, incorporating wildlife officials, customs and police officers from 28 countries, collaborated on investigation of >350 cases, leading to the arrest of ∼400 suspects.

### Corporate social responsibility and conservation practice

Another recent development in China is the rise of corporate social responsibility and contributions to biodiversity conservation. Roles have varied across different industries, but corporations have begun supporting a number of con- servation initiatives, including monitoring at-risk species and natural resources, establishing breeding centers, developing conservation breeding and reintroduction programs, creating charitable foundations to promote wildlife conservation, establishing green industrial supply chains to promote eco-friendly products and developing digital technological innovations to facilitate biodiversity conservation [6]. The Ant Forest project is a good illustrative model of corporate involvement in conservation, which has enlisted public participation to reduce their carbon footprint and support tree planting. By 2019, >500 million users had participated, resulting in 122 million trees being planted in arid and semi-arid areas in China. These achievements earned the program top honors for the United Nations’ environmental action category. In 2017, a consortium of 23 organizations including SEE Foundation launched the ‘Commonwealth Nature Reserve Alliance’ to enlist the public to protect 1% of the country's land area by 2030. By the end of 2019, a total of 39 PAs had been established, covering an area of 7630 km^2^, 0.079% of the country's land area.

### Community participation and traditional knowledge

Community-based biodiversity conservation initiatives are another area of growth in China. The public has been engaged through surveys of environmental beliefs and attitudes, engagement in the establishment of natural protection zones and citizen science, as well as broad environmental education initiatives. Traditional belief has also been enlisted in the service of biodiversity conservation. Community Conserved Areas (CCA), like Feng Shui Forests, capitalize on spiritual beliefs to protect sites with religious significance or mitigate exploitation of resources protected by religious or cultural taboos. These CCAs are typically small in size but may contain key habitats for endangered species, or serve as corridors connecting larger PAs facilitating wildlife dispersal or migration. Community engagement in these PAs leads to better protection within sacred mountains than outside [7].

## CHALLENGES AND PERSPECTIVES

### Top-level design of protected area

The early construction of PAs in China aims to rescue species from extinction. Although areas of PAs have greatly increased in past decades, the ecosystem integrity and structure connectivity are not well addressed. The lack of top-level design has resulted in problems such as spatial mismatch of PAs and wildlife, spatial overlap of different types of PAs, fragmentation and isolation of PAs and ineffective management [8]. Terrestrial nature reserves in China capture only 17.9% and 16.4% of the available habitat area for threatened mammals and birds, and even worse, 10.0% and 8.5% for amphibians and reptiles [9]. The marine PA network needs much more attention in both area and connectivity [10].

Currently, China is establishing a system of protected natural areas with national parks as its mainstay, supported by nature reserves as the foundation and complemented by various natural parks. Moreover, Ecological Conservation Redline, which is coordinated and aligned with other land-use-planning frameworks, is also under way. These top-level designs will integrate and optimize PAs that existed to cover more key areas for wildlife and ecosystem services, and expand to improve niche representation. In addition, the establishment of a marine national park is urgently needed to strengthen the protection of marine biodiversity [10]. Meanwhile other types of lands with conservation value, such as farmland, should be incorporated into protection plans [11]. Transboundary conservation reserves and corridors should be established for migratory species that traverse international borders such as the Amur tiger and leopard, the Chinese red panda, Asian elephant, the Myanmar snub-nosed monkey and the eastern black crested gibbon, to build a shared future for all life in transboundary areas.

### Science-based conservation and management

Lack of information about the demographic history, genetic diversity and adaptive strategies of endangered species has limited the development of conservation solutions and recovery actions. A notable counterexample is the giant panda, where science-informed policy and management contributed to downgrading of the species from ‘Endangered’ to ‘Vulnerable’ [12]. Therefore, future policy and management decisions should rest on the quality of the underlying science. A multidisciplinary scientific committee is needed to provide analysis, assessment and recommendations for decision-making, such as revising wildlife protection laws, setting conservation targets and formulating national conservation programs. Taxonomic changes among listed species should be addressed in a timely fashion, and lists of protected species require regular updating so that gaps can be closed, and that management interventions can be established for all species at risk. In addition, the science-based management of common species should be enhanced. With the implementation of national projects, and the absence of large- and medium-sized carnivores, the rapid growth of some herbivores, such as wild boar, is adversely affecting ecosystem stability. Scientific management intervention is required for species exceeding environmental carrying capacity.

### Long-term monitoring, information-sharing and evaluation

Although several wildlife monitoring networks are in operation, monitoring gaps and overlaps are common in practice, and indicators, methods and guidelines for regulating these networks are inconsistent because of the lack of top-level design. Most importantly, monitoring data cannot be shared fully, integrated and analyzed to guide management decisions and policy. In addition, although the terrestrial wildlife epidemic monitoring network, which is composed of 350 national, 768 provincial and many county-level monitoring stations has been established for >10 years, the capacity of wildlife epidemic control and monitoring is still insufficient.

To fill these gaps, further optimization and integration of current wildlife monitoring networks are needed to ensure that information can be freely shared. New technologies such as low-altitude remote sensing by unmanned aerial vehicles or small satellites, and thermal infrared remote sensing should be incorporated with traditional monitoring methods to obtain fine-scale integrated observations for better understanding of dynamic changes in wildlife and habitat. Besides species diversity, genetic diversity should also be monitored for the formulation of scientific and reasonable conservation strategy [13]. Given that wildlife-borne infectious disease is an important driver of species decline and extinction [14], new scientific frameworks such as updating current monitoring systems, establishing national key laboratories and genetic resource banks for detection of zoonotic diseases in the early stages are required. Furthermore, China has implemented many conservation programs; however, an indicator-based scientific index system to evaluate the effectiveness of these programs has not been established. Documenting outcomes of management interventions and protective measures on a regular basis is needed to guide the design and implementation of future conservation measures.

### Promote increased contribution by citizens and corporations

Citizen science and other forms of public engagement have great potential for raising public awareness, advancing scientific knowledge, and improving natural resource management and environmental protection [15]. Chinese policymakers, researchers and academics should make greater use of these forms to leverage public involvement in conservation science and practices, promoting the internalization of environmental ethics and cultivating ambassadors that champion conservation causes. CCAs are often more cost-effective compared with nature reserves because compliance with environmental stewardship comes from the community's attitudes rather than governmental regulation. The government should incorporate and promote this community-based approach and education initiatives whenever feasible. Further, China now has good models for corporate social responsibility and green practices, and these approaches should be promoted widely and adopted by other corporations. Corporate uptake of biodiversity conservation initiatives, awareness campaigns, green industrial chains and green products will be vital for the future of China's natural heritage.

Progress in wildlife conservation and management in China is founded on the establishment of a legal framework and the implementation of laws, regulations and science-based conservation programs during the past 40 years. Population recovery and reintroduction of some flagship species, and recent intensified efforts to combat illegal wildlife trade set a good precedent for others. However, the effective conservation and management of wildlife still presents challenges, many of which mirror challenges faced around the globe. China has the good fortune of a long historical legacy of human–nature coexistence in the philosophy of UNM. Mainstreaming this belief system is important to promote the urgently needed progress in biodiversity conservation. The Ecological Civilization building on this historical legacy provides an opportunity for the government to leverage people's beliefs to meet the conservation challenges articulated here. We believe that ancient Chinese beliefs have relevance today, and will help China and other countries to realize the harmonious coexistence of humankind and nature to build a shared future for all life on Earth.
